# Assessing Psychological Resilience: Development and Psychometric Properties of the English and Dutch Version of the Resilience Evaluation Scale (RES)

**DOI:** 10.3389/fpsyt.2018.00169

**Published:** 2018-05-15

**Authors:** Christianne A. I. van der Meer, Hans te Brake, Niels van der Aa, Pasha Dashtgard, Anne Bakker, Miranda Olff

**Affiliations:** ^1^Department of Psychiatry, Academic Medical Center, University of Amsterdam, Amsterdam, Netherlands; ^2^Impact, Dutch Knowledge and Advice Center for Psychosocial Care and Safety Concerning Critical Incidents, Diemen, Netherlands; ^3^Arq Psychotrauma Expert Group, Diemen, Netherlands; ^4^Foundation Centrum'45, Diemen, Netherlands; ^5^Department of Psychology and Social Behavior, University of California, Irvine, Irvine, CA, United States

**Keywords:** psychological resilience, scale development, traumatic stress, posttraumatic stress disorder (PTSD), measurement invariance, psychometric properties

## Abstract

**Background:** Psychological resilience is a distinct factor that affects mental health outcomes after adversities. This study describes the development, validity and measurement invariance (MI) of a Dutch and English scale on psychological resilience, called the Resilience Evaluation Scale (RES).

**Methods:** Separate online surveys with the Dutch and English version of the RES and hypothesized related measures were distributed in a Dutch- and English-speaking group, both drawn from the general population.

**Results:** Exploratory factor analysis, using data from 522 respondents (*n* = 296 Dutch, *n* = 226 English), yielded a two-factor structure for the final 9-item RES. The factors reflected the hypothesized underlying constructs of psychological resilience: *self-confidence* and *self-efficacy*. The items and constructs of psychological resilience as measured by the RES were interpreted and conceptualized in the same way by both language groups, with the exception of one item. The RES showed good convergent validity and good internal consistency.

**Conclusions:** The current study establishes sound psychometric properties of a new, brief, and freely available scale on psychological resilience. This study contributes to the identification and measurement of psychological resilience after adversities. The final 9-item RES may serve as a valuable instrument in research and in clinical practice.

## Introduction

An abundance of studies have shown a high prevalence of adverse outcomes such as posttraumatic stress, anxiety and depressive symptoms after potential traumatic events (PTE's) (1–3). However, more recently, there has been a shift in focus toward healthy adjustment and even thriving after experiencing trauma (4–9). Although a clear consensus on the definition is lacking (10), resilience is often defined as the process wherein an individual maintains a relatively stable, healthy level of psychological and physical functioning when confronted with PTE's (4, 11, 12). Resilience is influenced by multiple factors and varies in the context of individuals, environments, organizations and cultures (13). This complex and multidimensional nature of resilience makes it challenging to operationalize and measure the concept (8, 9, 13, 14). In order for resilience to establish itself as a meaningful concept in research and clinical practice, it is crucial to determine its distinct factors and to measure those factors in a reliable and valid way.

Factors that determine a resilient outcome after a PTE are often divided into two categories: *internal* capacities, and *external* factors (such as a healthy family environment) (13–17). A vital aspect of the internal capacity is psychological resilience, defined as the extent to which individuals evaluate themselves as being resilient (18, 19). According to the stress-coping model of Lazarus and Folkman (20), responses to stressful events are the result of the individual's appraisal of potential threat a situation imposes (primary appraisal) in combination with the individual's self-assessment of their own capability and resources to successfully handle the situation (secondary appraisal). It follows that an event will only be perceived as stressful when individuals believe that the demands of the situation exceed their coping abilities and resources (20). Secondary appraisal points at two potential underlying constructs of psychological resilience: *self-confidence* (i.e., trust in oneself) and *self-efficacy* (i.e., positive beliefs about adaptive coping with stressful situations) (21, 20). Aside from the external factors that determine a resilient outcome, internal capacities such as self-confidence and self-efficacy have been shown to be related to positive outcomes after stressful events, and buffer against symptoms of posttraumatic stress disorder (PTSD) (22–25).

Currently, several scales on resilience have been proposed (for a recent overview, see (10)). These scales often aim to assess both internal and external factors of resilience. For instance, the Connor-Davidson Resilience Scale (CD-RISC) (26) and the Resilience Scale for Adults (RSA) (27) aim to measure a composite of resilience factors, e.g., secure relationships, spiritual influences, family coherence, and social support. Indeed, various studies found conflicting conceptualizations (i.e., factor structures) for the CD-RISC across different populations and cultures, so it remains unclear which distinct resilience factors this scale measures (28–30). The same problems and findings regarding factor structure apply to the widely used Resilience Scale (RS) (31), which attempts to measure resilience factors such as perseverance, self-reliance, and meaningfulness (32–38). A clearer theoretical distinction between relevant resilience factors as well as a set of distinct measurement tools seems to be needed.

Moreover, despite the fact that most resilience scales are translated in different languages, it is often not investigated whether the concepts of the scales are measured in the same manner across different language groups (9). If this is the case, the scale is called measurement invariant across different language groups, which entails that the items of the scale, as well as the concepts they are measuring, are interpreted and conceptualized in the same way by individuals with different language backgrounds (39, 40). In order to meaningfully compare observed scale scores between language groups, it is essential that the scale is measurement invariant across those groups (40–42).

To address these gaps, the current study aims to describe the development, validation and measurement invariance (MI) of the first, brief, freely available measure for psychological resilience: the Resilience Evaluation Scale (RES). The RES is developed to operationalize psychological resilience as defined by the secondary appraisal of adverse events [cf. (20)]. The RES thereby focuses on the internal capacity in resilient behavior. The RES was developed in Dutch and English. This study examines the factor structure and psychometric properties of both versions of the RES, and whether the RES is measurement invariant across Dutch- and English-speaking groups. We expected that the RES would be measurement invariant across both language groups, and would be positively associated with a comparable measure of resilience. Also, we hypothesized that the RES would show positive associations with related constructs (i.e., self-efficacy, self-esteem, and level of global functioning), and a negative association with PTSD symptoms.

## Methods

### Development of the RES

The secondary appraisal concept of the Lazarus and Folkman model on stress guided the choice for a proposed two-factor RES: *self-confidence* and *self-efficacy*. These two factors were hypothesized to reflect internal psychological factors leading an individual to positively appraise its capacity to deal with an adversity. A team of clinicians and scholars with expertise in resilience and psychotrauma developed 10 new items for the Dutch version of the RES in several iterative sessions. The team did not use existing resilience scales in developing the 10 new RES items due to copyright regulations. In order to evaluate the RES, each team member filled out an evaluation form with open-ended questions on face validity, relevance, formulation, difficulty, and clarity for each RES item. In addition, overall suggestions or remarks about the RES and the RES instruction text were collected. The individual responses were then used in adapting the RES items, mostly in formulation, relevance and clarity. After multiple rounds of adaptations, re-evaluating the items within the team, and pilot testing with 10 trauma-exposed police officers, the Dutch version of the RES was established. Subsequently, the RES was translated by a certified translator (who fluently mastered Dutch and English) into English. Both versions were compared and evaluated within a team of Dutch- and English-speaking psychotrauma clinicians and scholars. Additional translation options were incorporated and back-translated by a psychotrauma expert who fluently mastered both Dutch and English. This back-translation was again evaluated within the team, and the best translation for each item was chosen. For this 10-item English version of the RES, see Table 1.

**Table 1:** Geomin rotated factor loadings for the two-factor solution model of the RES with 10 and 9 items as estimated by EFA.

	**2-factor solution 10 items**		**2-factor solution 9 items**	
	**F1**	**F2**	**F1**	**F2**
1. I have confidence in myself	0.215^*^	0.729^*^	0.214^*^	0.729^*^
2. I can easily adjust in a difficult situation	0.794^*^	−0.050	0.795^*^	−0.048
3. I am able to persevere	0.643^*^	0.032	0.643^*^	0.026
4. After setbacks, I can easily pick up where I left off	0.792^*^	0.043	0.784^*^	0.032
5. I am resilient	0.828^*^	−0.001	0.840^*^	0.001
6. I can cope well with unexpected problems	0.773^*^	−0.061	0.773^*^	−0.056
7. I appreciate myself	−0.040	0.907^*^	−0.039	0.907^*^
8. I can handle a lot at the same time	0.614^*^	0.029	0.611^*^	0.028
9. I believe in myself	0.007	0.916^*^	0.007	0.916^*^
10. I am not easily discouraged	0.418^*^	0.308^*^		

*Factor loadings greater than 0.60 are in bold. Model fit indices for the two-factor solution with 10 items: χ^2^ = 125.615, df = 26, CFI = 0.982, TLI = 0.969, RMSEA = 0.086. Model fit indices for the two-factor solution with 9 items: χ^2^ = 98.418, df = 19, CFI = 0.985, TLI = 0.971, RMSEA = 0.089; this model was selected as the model with the best factor solution*.

* *Significant at 5% level*.

Items 1, 3, 5, 7, and 9 of the RES were hypothesized to reflect the subscale *self-confidence*, and items 2, 4, 6, 8, and 10 were hypothesized to reflect the subscale *self-efficacy*. All items carry a 5-point range of responses: completely disagree (0), disagree (1), neutral (2), agree (3) and completely agree (4). The total score can be computed by summing the individual item scores, and varies from 0 to 40, with higher scores indicating greater psychological resilience.

### Procedure

This study was performed between 2014 and 2015. Separate online surveys were disseminated in an adult Dutch- and English-speaking group in the Netherlands and United States (U.S.) respectively. Both groups were drawn from the general (healthy) population. The surveys included demographic questions (age, gender, level of education and marital status), the 10-item RES, and a number of expected related constructs (see *Measures*). In the English-speaking group, the link to the survey was distributed via U.S. university online forums, social media and via personal social networks of an American researcher (author P. Dashtgard). The link to the Dutch online survey was distributed via newsletters, social media and e-mails to a network of social and behavioral scientists and clinicians, policy makers and college students. The links were accompanied by a short announcement about the purpose of the study and the target population (i.e., adults). The links were active for a period of 6 weeks in each country. Both surveys started with an introduction page which informed participants about the aim of the study and the estimated duration to complete the online survey. Also, contact details of the researchers were provided. In addition, all participants were informed that their answers on the online survey would be used in the study, and if they started the survey, they provided consent to use their data. Furthermore, participants were informed that all the data would be treated as strictly confidential, saved in a secured database, and that only the researchers would have access to this database. The study was conducted in compliance with the standard principles of ethical research established by the Academic Medical Center (AMC) and the principles of the Declaration of Helsinki. The Medical Ethical Committee of the AMC exempted this study from formal review because the Medical Research Involving Human Subjects Act (WMO) did not apply to the study (participants were asked to complete one online survey and the psychological integrity of the participants was not in question).

### Measures

The Dutch and English versions of the 25-item Resilience Scale (RS) were administered (31, 38). The RS is intended to measure the following five factors of resilience: equanimity (a balanced perspective of one's life and experiences), perseverance (being able to keep going despite difficulties), self-reliance (the belief in oneself and one's capabilities), meaningfulness (feeling that life has a purpose and life is valuable), and existential aloneness (sense of uniqueness, feeling of freedom). Following the rating instructions for the original versions of the RS, each item of the Dutch version is rated on a 4-point Likert scale (“1 = totally disagree” to “4 = totally agree”; total score ranging from 25 to 100) and each item of the English version is rated on a 7-point Likert scale (“1 = strongly disagree” to “7 = strongly agree”; total scores ranging from 25 to 175). Higher scores indicate greater resilience.

The Rosenberg Self-Esteem Scale (RSE) measures an individual's overall sense of being worthy as a person (43, 44). In both versions, the 10 items are rated on a 4-point Likert scale (“0 = strongly agree” to “3 = strongly disagree”). Higher scores reflect more self-esteem.

The General Self-Efficacy Scale (GSE) (45) is a 10-item questionnaire that measures optimistic self-beliefs to cope with stressful situations (45–47). Each item is rated on a 4-point Likert scale, ranging from “1 = completely incorrect” to “4 = completely correct” for the Dutch version, and “1 = not at all true” to “4 = exactly true” for the English version. Higher scores reflect greater general self-efficacy.

Respondents were asked to rate their current level of global functioning, considering both private life and work, on a 10-point Likert scale (“1 = extremely bad” to “10 = excellent”).

For the Dutch-speaking sample, the 10-item Trauma Screening Questionnaire (TSQ) (48, 49) was used to screen for PTSD symptoms (re-experiencing and arousal symptoms). For each item, participants indicate whether or not (a score of 1 or 0 respectively) they had experienced the particular symptom at least twice in the past week. The total score ranges from 0 to 10, with higher scores indicating more PTSD symptoms. Since DSM-5 PTSD screening measures were available at the time we conducted our study in the English-speaking sample, the PTSD checklist for DSM-5 (PCL-5) was used (50). The PCL-5 is a self-report questionnaire assessing the 20 PTSD symptoms in the past month and the following symptom clusters: re-experiencing; avoidance; negative alterations in cognitions and mood; and alterations in arousal and reactivity. Each item is rated on a 5-point Likert scale (“0 = not at all” to “4 = extremely”), resulting in a total score from 0 to 80, with higher scores reflecting more PTSD symptoms. The TSQ and PCL-5 were only administered if respondents indicated that they had experienced a PTE.

In the current study, the internal consistency of all the above-mentioned measures ranged from good to excellent. See Table 2 for the Cronbach's α found in this study for all the administered measures.

**Table 2:** Spearman's rho correlations between the RES (total and subscale scores) and the positively and negatively related scales (total scores) and the internal consistency for all administered measures.

	**RES total**		**RES self-efficacy**		**RES self-confidence**		**Cronbach's** α	
	**Dutch**	**English**	**Dutch**	**English**	**Dutch**	**English**	**Dutch**	**English**
Resilience (RS)	0.62^**^ (*n* = 274)	0.74^**^ (*n* = 200)	0.54^**^	0.66^**^	0.51^**^	0.65^**^	0.843	0.909
Self-efficacy (GSE)	0.55^**^ (*n* = 257)	0.73^**^ (*n* = 162)	0.51^**^	0.70^**^	0.42^**^	0.58^**^	0.856	0.857
Self-esteem (RSES)	0.53^**^ (*n* = 256)	0.71^**^ (*n* = 155)	0.36^**^	0.53^**^	0.64^**^	0.81^**^	0.885	0.917
Global functioning^a^	0.47^**^ (*n* = 214)	0.55^**^ (*n* = 226)	0.37^**^	0.52^**^	0.39^**^	0.46^**^	–	–
PTSD symptoms: (TSQ or PCL-5)^b^	−0.22^**^ (*n* = 171)	−0.39^**^ (*n* = 77)	−0.20^**^	−0.26^*^	−0.16^*^	−0.47^**^	0.802	0.958

*RES total, total score item 1–9; RES self-efficacy, total score of items 2, 3, 4, 5, 6, and 8; RES self-confidence, total score of items 1, 7 and 9. All depicted correlation coefficients are Spearman's rho correlation coefficients*.

* *p < 0.05*,

** *p < 0.01*.

a *This item was placed as the second item in the English survey, and as the last item in the Dutch survey. There was missing data (n = unknown) on this item in the Dutch survey due to a technical error*.

b *The TSQ was administered in the Dutch-speaking sample, the PCL-5 was administered in English-speaking sample (only to respondents that indicated that they had experienced a stressful event in their life)*.

### Statistical analyses

Differences in respondent characteristics between the two language groups were assessed by conducting Mann-Whitney *U*-tests (non-normally distributed variables), chi-square tests (categorical variables), or Fisher's exact tests (categorical variables with cell frequencies <5), using SPSS Version 23.

To determine the factor structure of the RES in the total sample, an *exploratory factor analysis* (EFA) with geomin rotation for ordinal data with the WLSMV estimator was conducted in Mplus version 7.3 (51). The WLSMV estimator is recommended in factor analytic procedures with categorical data (51). Exploratory factor analysis (EFA) was used to test the factor structure of the items of the RES. EFA was deemed the most useful strategy for this purpose because the RES is a novel measure for which new items were constructed which were not based on the items of existing scales. In addition, the RES is the first measure of psychological resilience. Therefore, no previously defined factor model for psychological resilience could be used and it should first be tested whether the constructed items were interpreted and conceptualized in the intended way. Due to low frequency of the first response category (i.e., “0 = completely disagree”) on some items of the RES, the two lowest response categories were merged into one. An underlying normal distribution was assumed for each item, where the resulting four response categories were divided by three thresholds which were estimated from the data. Five models with one to five factor solutions were examined. Multiple parameters, i.e., Kaiser criterion (eigenvalue >1) and the model fit statistics comparative fit index (CFI), Tucker-Lewis Index (TLI), and root mean-square residual error of approximation (RMSEA) were used to assess the number of latent factors needed to adequately account for the correlations among item scores. The model with the optimal balance between model fit, parsimony, and conceptual interpretability was selected as the best factor solution.

*Measurement invariance* (MI) of the RES across the two language groups was examined in Mplus version 7.3 (51). Three levels of MI (i.e., configural, scalar and strict measurement invariance) were investigated by conducting a typical sequence of single and multigroup factor models using confirmatory factor analyses (CFA) with categorical factor indicators (40, 52, 53). In factor analytic models with categorical data, metric measurement invariance cannot be tested because factor loadings and thresholds can only be constrained in tandem (51). Factor models were estimated with the WLSMV estimator using the THETA parameterization. The first level of MI, *configural invariance*, implies that the underlying construct is conceptualized in a similar manner by respondents from different groups (42). Configural invariance is met when the same factor structure is valid across groups, but parameter estimates (i.e., factor loadings, thresholds, and residual variances) may vary across groups. Configural invariance was tested by fitting the best factor model derived from the EFA in a multiple group CFA for the total sample, wherein the factor loadings and thresholds were freely estimated across the language groups, and the residual variances were fixed at one in both groups. In addition, single group CFA's were fit for the two language groups separately. The second level of MI, *scalar invariance*, entails that the strength of the relations between the items and the underlying construct is similar across groups, i.e., that individuals in different groups attribute the same meaning to the construct under study (42), and that cross-group comparisons of mean differences on the underlying construct are meaningful. Scalar invariance is met when, in addition to configural invariance, factor loadings and thresholds are equal across groups (40, 42), and was tested by fitting a multigroup CFA in which factor loadings and thresholds were constrained to be equal across groups, and the residual variances were fixed at one in the first group and freely estimated in the second group. The model fit of scalar invariance was compared with the model fit of the multigroup CFA representing configural invariance. When all thresholds and factor loadings are invariant across groups, scalar invariance holds. In case it does not hold, cross-group comparisons of latent (i.e., not observed) mean differences are still meaningful as long as scalar MI holds for at least two items (54). When scalar invariance does not hold, *partial scalar invariance* should be examined by studying the largest differences in thresholds and factor loadings between groups (40, 42). Partial scalar invariance was tested by releasing the constraints for the item with the largest between-group differences in thresholds and factor loadings, determined by scrutinizing the largest modification indices in the scalar invariance model. The model fit of partial scalar invariance was compared to the model fit of configural invariance.

Model fit of the single and multigroup CFAs was evaluated with the CFI, TLI, and RMSEA. For CFI and TLI, model fit is considered good if values are close to or larger than 0.95 (55). For the RMSEA, a value <0.05 indicates good model fit, a value between 0.05 and 0.08 suggests adequate model fit, a value between 0.08 and 0.10 indicates a mediocre model fit, and >0.10 indicates a poor fit (56). To compare the goodness-of-fit between the nested MI models, the χ^2^ difference test and the difference in CFI values (< 0.01) between nested models were used (57). The “difftest” option in Mplus was used for appropriate χ^2^ difference testing with the WLSMV estimator (51). Because the χ^2^ difference test is highly sensitive to sample size, it may reject models that actually fit the data (40, 57). It is recommended to interpret the χ^2^ difference test by the ratio of the χ^2^ value and the degrees of freedom (χ^2^/df ratio). The nested model has a better fit than the more complex model if the ratio is less than 3 (58).

*Convergent validity* of the RES was examined by calculating the correlations between the RES total and subscale scores (as derived from the EFA) and the total scores of the following expected positively related measures: RS (resilience), RSES (self-esteem), GSE (general self-efficacy), and a single item measuring global functioning in the two languages groups separately. In addition, the total and subscale scores of the RES were correlated to the hypothesized negatively related total scores of the TSQ and PCL-5 (PTSD symptoms in the Dutch and English samples respectively). Because the RES scores followed a non-normal distribution, Spearman's rho correlations were used.

The *internal consistency* of the RES was assessed by obtaining the inter-item and item-total correlations, the Cronbach's α, and alpha if item deleted for the RES total scale and the two subscales (as derived from the EFA) for the two language groups separately. A Cronbach's α of ≥0.90 indicates excellent internal consistency, a value between 0.90 and 0.80 reflects good internal consistency (59).

## Results

### Respondent characteristics

In total, 569 individuals responded to the online survey, of whom 92% (*n* = 524) completed the RES. The scores of two respondents were excluded due to an unreliable response pattern (in both cases, the same answer was given on all the items of different scales). Table 3 presents the respondent characteristics and RES scores for the total sample (*n* = 522), and the Dutch (*n* = 296) and English (*n* = 226) subsample separately. The English group consisted of more female respondents than the Dutch group [$\chi_{\left(1\right)}^{2}$ = 8.973, *p* < 0.05]. The English group was younger (U = 19121.00, *p* < 0.001), higher educated [$\chi_{\left(1\right)}^{2}$ = 12.535, *p* < 0.001] and more often single than the Dutch group [*F*_(4)_ = 63.551, *p* < 0.001, Table 3]. The two language groups did not significantly differ in terms of the mean score on the total RES scale and the mean score on the RES subscale self-efficacy. The Dutch group had a higher mean score on the RES subscale self-confidence than the English group (*U* = 29932.00, *p* < 0.05).

**Table 3:** Respondent characteristics and RES scores.

	**Total group (*n* = 522)**	**Dutch group (*n* = 296)**	**English group (*n* = 226)**	**Test-value**	***p***
Female, *n* (%)	371 (71.1)	195 (65.9)	176 (77.9)	$\chi_{\left(1\right)}^{2}$ = 8.973	<**0.05**
Age, mean (SD)	37.2 (13.04)	41.9 (13.7)	31 (8.9)	U = 19121.00	<**0.001**
Education, *n* (%)^*^				$\chi_{\left(1\right)}^{2}$ = 12.535	<**0.001**
Low	71 (13.6)	54 (18.2)	17 (7.5)		
High	451 (86.4)	242 (81.8)	209 (92.5)		
Marital status, *n* (%)				*F*_(4)_ = 63.551	<**0.001**
Single	207 (39.7)	75 (25.3)	132 (58.4)		
Married/cohabitation	268 (51.3)	188 (63.5)	80 (35.4)		
Divorced	26 (5)	15 (5.1)	11 (4.9)		
Widow(er)	7 (1.3)	7 (2.4)	0 (0)		
Other	14 (2.7)	11 (3.7)	3 (1.3)		
**RES**					
Total score	25.76 (5)	25.95 (4.11)	25.52 (5.98)	U = 33043.50	0.81
Subscale self-efficacy	17.29 (3.48)	17.24 (2.98)	17.36 (4.05)	U = 31233.00	0.19
Subscale self-confidence	8.47 (2.18)	8.71 (1.80)	8.16 (2.57)	U = 29932.00	<**0.05**

*RES total, total score item 1–9; RES self-efficacy, total score of items 2, 3, 4, 5, 6, and 8; RES self-confidence, total score of items 1, 7, and 9*.

* *High level of education included higher professional education, university and graduate school. Low level of education included elementary, primary, middle and high school, lower and secondary professional education. p-values < 0.05 are in bold*.

### Exploratory factor analysis

The EFA on the total sample yielded a two-factor solution as a good fit (based on the eigenvalues, CFI, TLI, and RSMEA values) for the 10 RES items with eigenvalues of 5.545 and 1.217 respectively. Eigenvalues of the third to tenth factor were all lower than one (0.696, 0.656, 0.565, 0. 383, 0.348, 0.231, 0.191 and 0.167 respectively).

The CFI and TLI values of the two-factor solution indicated a good model fit, the RSMEA value reflected a mediocre model fit (Table 1). Item 10 cross-loaded significantly on both factors, with only a small difference between the two factor loadings (λ = 0.110), indicating that item 10 did not sufficiently distinguish between both factors (Table 1). Also, item 10 showed relatively low factor loadings on the first and second factor (λ = 0.418 and λ = 0.308 respectively), suggesting that this item did not considerably add to either factor. Therefore, the EFA was rerun without item 10, yielding a two-factor solution with eigenvalues of 5.098 and 1.210 respectively (eigenvalues of the third to tenth factor were lower than one, ranging from 0.169 to 0.670). The CFI and TLI indicated a good model fit, the RSMEA indicated a mediocre model fit (Table 1). All factor loadings on the two factors were significant and no cross-loadings were observed. This model, without item 10, was selected as the best factor solution. Factor 1 (items 2, 3, 4, 5, 6, 8) was termed “self-efficacy” and factor 2 (items 1, 7, 9) was labeled “self-confidence.” For the final 9-item version of the RES, see Appendix A.

### Measurement invariance

The two-factor solution with 9 items derived from the EFA was used for the MI analysis across the two language groups. Table 4 presents the details of the performed models and model fitting results for each level of MI. In model 1, the CFI and TLI indicated good model fit, and the RMSEA suggested adequate model fit. In model 1a and 1b, the CFI and TLI also indicated good model fit, and the RMSEA a mediocre and acceptable model fit for the English and Dutch group respectively. Therefore, configural invariance is met for the RES across the two language groups. In model 2, the CFI and TLI represented a good model fit, and the RMSEA indicated adequate model fit. Although the difference in CFI between model 1 and 2 was acceptable, the χ^2^/df ratio between model 1 and 2 suggested a worse fit of model 2 compared to model 1. Consequently, full scalar invariance did not hold, and partial scalar invariance was examined by studying potential between-group differences in thresholds and factor loadings. The modification indices indicated a substantial between-group difference in the thresholds of item 4. Model 3 tested a multigroup two-factor model, where thresholds and factor loadings were constrained to be equal across groups, except for item 4. The CFI and TLI of model 3 suggested a good model fit, and the RMSEA indicated adequate model fit. The χ^2^/df ratio and the difference in CFI indicated that the fit of model 3 was not worse compared to model 1. Model 3 was preferred over model 1 and 2, indicating that partial scalar invariance holds for the RES across the two language groups (Table 4).

**Table 4:** Model fitting results for each measurement invariance level of the RES across the English- and Dutch- speaking groups.

	**vs**.	**χ^2^**	**df**	***Δχ*^2^**	**Δdf**	**χ^2^/df**	**CFI**	**ΔCFI**	**TLI**	**RMSEA**
1. Configural invariance: total sample	–	125.224	52	–	–	–	0.987	–	0.981	0.073
1a. Configural invariance: English language	–	68.352	26	–	–	–	0.987	–	0.981	0.085
1b. Configural invariance: Dutch language	–	57.336	26	–	–	–	0.986	–	0.981	0.064
2. Scalar invariance	1	197.990	75	75.614	23	3.29	0.977	−0.010	0.978	0.079
**3. Partial scalar invariance**	**1**	**171.357**	**72**	**49.483**	**20**	**2.47**	**0.982**	–**0.005**	**0.982**	**0.073**

*Model 1: multigroup two-factor model with free estimation of thresholds and factor loadings across groups. Model 1a and 1b: two-factor model with free estimation of thresholds and factor loadings for each language group. Model 2: multigroup two-factor model with thresholds and factor loadings constrained to be equal across groups. Model 3: multigroup two-factor model with thresholds and factor loadings constrained to be equal across groups, except for item 4. The model with the best model fitting indices is printed in bold, partial scalar invariance holds. vs. = versus (the model of comparison). χ^2^, df = chi-square test statistic value and degrees of freedom. Δχ^2^, Δdf = chi-square test statistic value and degrees of freedom for chi-square difference test between two nested models. χ^2^/df = ratio between χ^2^ and degrees of freedom for the chi-square difference test. ΔCFI = CFI difference between two nested models*.

### Convergent validity

Table 2 presents the correlations between the 9-item RES (total and subscale scores) and all hypothesized related questionnaires (total scores). There was a significant positive correlation between the RES scores and all hypothesized related constructs (i.e., resilience, self-efficacy, self-esteem, and global functioning) in both language groups (Table 2). The RES total score showed the highest positive correlation with the RS total score (resilience) in both groups. Also, a significant negative correlation was found between the RES total and subscale scores, and the total PTSD symptom scores in both groups.

### Internal consistency

Table 5 presents the internal consistency measures (Cronbach's alpha and the inter-item and item-total correlations) for the 9-item RES. Cronbach's alpha for the 9-item RES total and subscale scores was good in both language groups, and acceptable for the subscale *self-efficacy* in the Dutch group. Cronbach's α did not improve if items were deleted (this applied to the RES total scale and subscales in both groups).

**Table 5:** Internal consistency of the RES (total score and subscale scores).

	**Cronbach's** α		**Range inter-item correlation**		**Range item-total correlation**	
	**Dutch**	**English**	**Dutch**	**English**	**Dutch**	**English**
**RES**						
Total score	0.825	0.898	0.122–0.665	0.318–0.751	0.370–0.653	0.539–0.738
Self-efficacy subscale	0.786	0.870	0.197–0.601	0.367–0.661	0.407–0.645	0.533–0.766
Self-confidence subscale	0.838	0.887	0.607–0.665	0.682–0.751	0.678–0.723	0.762–0.817

*RES total = total score of item 1–9; RES self-efficacy = total score of items 2, 3, 4, 5, 6, and 8; RES self-confidence = total score of items 1, 7, and 9*.

## Discussion

This study demonstrates a two-factor structure and sound psychometric properties of a new, brief, freely available Dutch and English scale on psychological resilience, called the RES. The final 9-item RES consists of two underlying constructs of psychological resilience: *self-confidence* and *self-efficacy*. The Dutch- and English-speaking group interpreted and conceptualized the items and underlying constructs of the RES in the same manner, with the exception of one item. Furthermore, the RES showed good convergent validity, and good internal consistency.

The RES was developed to operationalize psychological resilience, and the secondary appraisal concept of the Lazarus and Folkman model on stress guided the choice for a proposed two-factor RES (i.e., *self-confidence* and *self-efficacy*), assessing psychological resilience. Indeed, EFA yielded the best factor solution for a model with two factors that reflected these hypothesized constructs. Item 10 “I am not easily discouraged” was removed from the scale because it did not substantially add to, and insufficiently differentiated between, both factors. Item 10 was a Dutch expression, with no direct English equivalent available. Therefore, the item potentially reflected different concepts in both versions of the RES, which in turn may have led to a different conceptualization of the item between the Dutch- and English-speaking group. Also, item 10 was the only item with a negation, which could increase the chance of misinterpretation (60).

A two-factor structure was found for the final 9-item version of the RES, with three items reflecting *self-confidence* and six items clustering on *self-efficacy*. Contrary to our hypothesis, item 3 “I am able to persevere” and item 5 “I am resilient” clustered on the construct *self-efficacy* instead of *self-confidence*. On a conceptual level, being resilient and able to persevere could be interpreted as more closely related to behavior during difficulties, and therefore adaptive coping, rather than to a general positive belief in oneself, i.e., self-confidence (61). Also, based on face validity, the three RES items that were intended to and found to reflect *self-confidence* (“I have confidence in myself,” “I appreciate myself,” and “I believe in myself”) seem to capture the construct *self-confidence* in a more direct and literal manner than item 3 and item 5. Furthermore, it should be noted that differentiating between the two constructs of psychological resilience is somewhat ambiguous because it is likely that beliefs of *self-confidence* and *self-efficacy* within psychological resilience are conceptually related, which may lead to challenges in strictly distinguishing the two constructs. This may potentially have contributed to the finding that two items clustered on the construct *self-efficacy* instead of *self-confidence*. Future work on the factor structure of the RES needs to further establish and replicate the current structure, as well elucidate as how these psychological resilience factors relate to or distinguish from other proposed resilience factors.

The MI analysis showed that the RES is partial scalar invariant across the two language groups. This means that the items and the constructs of psychological resilience as measured with the RES are interpreted and conceptualized in the same manner by individuals with a Dutch or English language background, with the exception of item 4 (“After setbacks, I can easily pick up where I left off”). This implies that cross-group comparisons of observed scale scores (i.e., result of summing the individual item scores) with regard to the RES partly reflect measurement bias instead of true underlying differences. Therefore, cross-group comparisons of observed scale scores are only meaningful when item 4 is discarded. This finding provides a great opportunity to compare the RES scores (without item 4) between Dutch and English groups on a global level, in research and in clinical practice. Interestingly, similar to item 10, item 4 is a Dutch saying, which could have led to differences in conceptualization and interpretation of the item between the two languages groups. The other eight items of the RES do not reflect a Dutch saying and had direct English equivalents.

The RES demonstrated good convergent validity. In both language groups, the RES total scale and subscales were positively associated with all the hypothesized related measures (i.e., resilience, self-esteem, self-efficacy, and global functioning). Furthermore, the confirmed negative correlation between the RES and PTSD symptoms in both language groups concurs with the previous finding that resilience buffers against PTSD symptoms (22–25). Of notice, the convergent validity and internal consistency values were higher in the English speaking group than in the Dutch speaking group. Further research is needed to shed some insight into potential explanations for this result.

Some limitations of the current study should be considered. Our sample consisted mostly of women and highly educated individuals, limiting generalizability to the general Dutch- and English-speaking population. Replication in other study samples is warranted with regard to a variety of sample characteristics, such as gender, education, language, culture, (high-risk) profession, and mental health status. A different PTSD measure was used in the Dutch- and the English-speaking group, limiting the comparison between groups on the relationship between psychological resilience and PTSD symptoms. Results on other specific psychometric properties of the RES such as the short- and long-term test-retest reliability, the robustness and stability of the RES scores, the relation between the RES and theoretically related scales such as the RSA and CD-RISC, and the discriminant validity of the RES are not investigated in this study, and should be examined in future research. A full structural equation modeling approach would be an interesting method in this regard, to simultaneously study the measurement model for the RES and the relationships with hypothesized related constructs. Also, longitudinal studies in healthy individuals, as well as in patients with psychological problems, could provide information on the sensitivity of the RES in capturing changes in psychological resilience over time, as well as the characteristics of the scale in observing reliable clinical change. Prospective longitudinal designs may broaden our understanding of the mechanisms underlying resilient outcomes, and the predictive validity of the RES on mental health after adversities in general. To note, the final 9-item RES is currently used and examined as one of the outcome measures in a randomized controlled trial on the effectiveness of a PTSD self-help tool in traumatized individuals (62). In addition, a Chinese translation of the RES is developed and the psychometric properties of this scale are currently investigated (63).

The current study has several important strengths. Studies on the MI of resilience scales are seriously lacking (9, 32), this study fills this gap by extensively investigating MI in two language groups. Further, our sample was relatively large and the completion rate of the RES was very high, the latter reducing potential biases. Also, we used valid and reliable instruments to determine the validity of the RES. Moreover, the convergent validity of the RES was examined in the two groups separately (serving as replication samples), which strengthened the results regarding construct validity.

To conclude, the final 9-item RES is a valid, reliable, and valuable instrument that can be used in its current form on a large scale worldwide, both in research and in clinical practice. This study contributes to the urgent need for identifying and measuring distinct factors that affect mental health outcomes after trauma. The final 9-item RES can be freely used on a global level by individuals with a Dutch or English language background, and cross-group comparisons on the observed scores are meaningful (when item 4 is discarded). Scholars and clinicians are encouraged to use the final 9-item RES in other populations and research designs, hopefully replicating our finding that psychological resilience is a distinguishable construct, and strengthening the universal use of the RES. By giving ample attention to translation, adaptation and cross-cultural validation (as was done in this study), it will deepen our understanding of the factors that play a role in resilience and its potential determinants.

## Author contributions

CvdM, HtB, AB, and MO designed the study. PD commented on the design of the study and collected data in the U.S. sample. HtB and CvdM collected data in the Dutch sample. CvdM and NvdA performed the statistical analyses. All authors commented on the statistical analyses. CvdM wrote the first draft of the manuscript and prepared the tables. All the authors contributed to the manuscript, and have approved the final version of the manuscript.

### Conflict of interest statement

The authors declare that the research was conducted in the absence of any commercial or financial relationships that could be construed as a potential conflict of interest.

## Appendix A:

### Final 9-item english version of the resilience evaluation scale (RES)

RES instruction: Below you will find a number of statements about how you think about yourself and the way in which you usually respond to difficult situations. Please indicate to what extent each statement applies to you.

**Table:**

	**Completely disagree**	**Disagree**	**Neutral**	**Agree**	**Completely agree**
1. I have confidence in myself	0	1	2	3	4
2. I can easily adjust in a difficult situation	0	1	2	3	4
3. I am able to persevere	0	1	2	3	4
4. After setbacks, I can easily pick up where I left off	0	1	2	3	4
5. I am resilient	0	1	2	3	4
6. I can cope well with unexpected problems	0	1	2	3	4
7. I appreciate myself	0	1	2	3	4
8. I can handle a lot at the same time	0	1	2	3	4
9. I believe in myself	0	1	2	3	4
